# Eco‐Friendly, Sound Absorbing Materials Based on Cellulose Acetate Electrospun Fibers/*Luffa Cylindrica* Composites

**DOI:** 10.1002/marc.202400863

**Published:** 2024-12-27

**Authors:** Viktoria Theodorou, Michal Matysík, Iveta Plšková, Ivo Kusák, Petri Ch. Papaphilippou, Theodora Krasia‐Christoforou

**Affiliations:** ^1^ Department of Mechanical and Manufacturing Engineering University of Cyprus 1 Panepistimiou Avenue Nicosia Aglantzia 2109 Cyprus; ^2^ Faculty of Civil Engineering Institute of Physics Brno University of Technology Veveří 331/95 Brno 602 00 Czech Republic

**Keywords:** cellulose acetate fibers, electrospinning, *Luffa Cylindrica*, sound absorption, standing wave ratio method

## Abstract

Sound absorption plays a crucial role in addressing noise pollution that may cause harm to both human health and wildlife. To tackle this environmental issue, the implementation of natural‐based sound absorbing materials attracts considerable attention in the last few years. In this study, sound absorbing, eco‐friendly composites are produced by combining a 3D natural sponge namely *Luffa Cylindrica* (LC) with cellulose acetate (CA) microfibrous layers that are fabricated through electrospinning. Electrospun microfibers can effectively absorb sound waves due to their unique properties such as high porosity, small diameter, and large surface area. The individual components and the resulting composites, exhibiting various configurations, are characterized in respect to their morphology, porosity, density, and sound absorption properties. More precisely, the sound absorption coefficient is determined through the standing wave ratio method within the range of 500–4000 (Hz) frequency. The most promising materials consist of a multilayer combination of LC with CA microfibrous layers, which creates new prospects in the development of such materials for sound absorption applications.

## Graphical abstract

A 3D natural sponge named *Luffa Cylindrica* (LC) and cellulose acetate (CA) microfibrous layers fabricated through electrospinning are combined in various configurations, showing potential as innovative, eco‐friendly solutions for sound absorption applications.

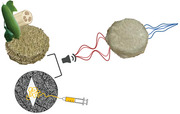

## 1 Introduction

Prolonged exposure to high levels of noise can cause Noise Induced Hearing Loss (NIHL), sleep disturbances, psychophysiological, and mental health problems. Beyond its impact on individuals, noise pollution can disrupt ecosystems and interfere with communication among species.^[^ ^1^ ^]^ Therefore, it is necessary to create an acoustically desirable environment safeguarding the protection of the physical and mental health. The importance of utilizing materials with sound absorbing properties lies in their ability to create that healthier environment and ensuring the sustainability of both urban and natural spaces. Generally, sound absorption materials can be classified as either porous or resonant materials.^[^ ^2^ ^]^ Resonant materials exhibit a high sound absorption coefficient value at low frequencies via a local vibration resonance effect.^[^ ^3^ ^]^ In comparison with the resonant materials, porous materials present good sound absorption performance at high frequencies.^[^ ^3^ ^]^ The number, size and type of pores are important factors that can affect sound absorption. When sound waves propagate across the surface of a material, the interaction between the material and the air molecules found within the pores produces friction. Consequently, the sound energy undergoes a transformation into thermal energy, and it is dissipated due to both viscous and thermal effects.^[^ ^4^ ^]^

Porous materials are commonly utilized as sound absorbers in noise control applications. These materials consist of a variety of substances, including glass wool, minerals, and agricultural‐based foams and fibers.^[^ ^5^ ^]^ More recently adopted organic alternatives include among others polyurethane (PU), melamine and polystyrene foam.^[^ ^1^ ^]^ Although inorganic materials originate from natural minerals, their extraction and processing pose significant health risks along with environmental concerns. Similarly, organic materials, derived from petrochemical sources, contribute to environmental degradation due to fossil fuel extraction and associated carbon emissions. Both types of materials are challenging to recycle, are not biodegradable, and can often end up in landfills. Consequently, there is an urgent need to develop environmentally friendly alternatives to replace conventional sound absorbing materials. The use of natural fibers has the potential to assist in the transition toward sustainable and green development, while introducing lightness, high porosity, biodegradability, and design flexibility.^[^ ^1^, ^6^ ^]^

Structural design is an effective approach to enhance the sound insulation properties of fiber composites.^[^ ^7^, ^8^ ^]^ Bilayer or multilayer plate insulation structures are employed to maximize the reduction of acoustic energy. Previous research work has demonstrated that multiple reflections of sound waves and various forms of interlayer deformation during wave propagation through multilayered materials can effectively dissipate acoustic energy.^[^ ^9^ ^]^

Viscoelastic polymer materials are commonly used as interlayers in sandwich structures to enhance sound transmission loss, due to their excellent damping properties. Yoon et al.^[^ ^10^ ^]^ demonstrated that by incorporating PU as a constrained layer between two steel plates the transmission loss of the composite is significantly improved. Similarly, multilayered materials have been simulated and fabricated to boost sound insulation performance. Studies have shown that the multiple reflections of sound waves and various interlayer deformations during wave propagation in multilayered materials are effective in dissipating acoustic energy.

Li et al.^[^ ^11^ ^]^ conducted numerical analyses on wave propagation in composites comprised of alternating layers of pure polymer and polymer reinforced with carbon nanofiber sheets. Their findings revealed that by adjusting the number of layers and the thickness of each layer wave propagation can be influenced, and the transmission coefficient can be controlled. These studies collectively suggest that multilayered structures are highly effective in enhancing the sound insulation properties of composites.

Multilayer nonwoven composite structures have been utilized to enhance acoustic performance. In multilayer polyester composites, densely packed layers contribute to improved sound absorption properties.^[^ ^12^ ^]^ Coconut coir fiber has been shown to exhibit a higher sound absorption coefficient when compressed into bales or mattress sheets. Compared to a single layer, multilayer coconut coir fibers with air gaps between the layers enhance the material's absorption coefficient, particularly at lower frequencies.^[^ ^13^ ^]^ Sandwich structures made of polylactide (PLA)/hemp/polylactide, and polypropylene (PP)/glass fiber/polypropylene have demonstrated improved sound absorption in mid to high frequency ranges.^[^ ^14^ ^]^

In another study,^[^ ^15^ ^]^ multilayered composite plates were fabricated using layers of composite materials combined with commercially available materials such as cork or felt. The composite materials consisted of fir sawdust and PU foam, demonstrating effective sound absorption across a broad frequency range, particularly when measurements were taken on the sawdust side. Incorporating cork as a surface layer enhanced sound absorption in the 100–1400 Hz frequency range but reduced it at higher frequencies.

Electrospun micro‐ and nanofibers combined with porous materials can efficiently enhance sound absorption, particularly at medium and high frequencies.^[^ ^16^ ^]^ The fibers deriving from the electrospinning process are solid, continuous, and can be employed in a variety of applications including sound dampening and protective clothing,^[^ ^17^ ^]^ tissue engineering,^[^ ^18^ ^]^ filtration,^[^ ^19^, ^20^ ^]^ energy,^[^ ^21^, ^22^ ^]^ and sensing.^[^ ^23^ ^]^ Electrospun nanofibers offer several appealing characteristics including cost‐effectiveness, energy efficiency, and a combination of high‐surface area and high rate of absorption.

Electrospinning is a widely employed technique for producing sound absorbing materials.^[^ ^24^, ^25^, ^26^ ^]^ In one such example, electrospinning was employed to synthesize nanofibrous membranes consisting of interwoven webs of nanofibers with high porosities. These membranes were combined with a melamine foam substrate to form a component of a bionic sound absorber.^[^ ^27^ ^]^ In another work, the authors demonstrated that the use of electrospun polyvinylidene fluoride membranes, doped with graphene, can improve sound‐energy absorption, particularly in the middle frequency range.^[^ ^28^ ^]^ Overall, this method allows the adjustment of various factors, including the chemical composition of the materials used, the morphology of the produced fibers and the thickness of the produced electrospun fibrous mats that can be tuned by altering the electrospinning duration.^[^ ^26^ ^]^

Based on previous studies, it can be concluded that multilayered structures are effective in improving the sound insulation properties of composites. In our view, the multilayered structure of cellulose acetate (CA) fibrous mats and *Luffa* *Cylindrica* (LC) holds significant potential for further enhancing of sound insulation performance.

Herein, composite materials comprised of CA electrospun fibrous mats and LC were prepared and further evaluated in respect to their sound absorption performance. CA (that is a cellulose derivative) was chosen due to its cost‐effectiveness, biodegradability, and lower environmental impact compared to synthetic materials.^[^ ^29^ ^]^ LC is a 3D natural fibrous sponge classified in the cucumber family. It presents a unique morphology consisting of multidirectional pores and it demonstrates high toughness and good mechanical strength.^[^ ^30^ ^]^ LC was selected to be used in the present study as the main, naturally‐derived structural component, due to its distinctive tubular structure that can affect the sound absorption coefficient.^[^ ^4^, ^31^ ^]^ In combination with electrospun CA fibrous mats in various configurations, the produced eco‐friendly composites were evaluated in respect to their sound absorption properties by employing the standing wave ratio method, demonstrating that such multi‐layer naturally derived configurations could be appropriate in the development of highly efficient sound absorbing materials.

## 2 Experimental Section

### 2.1 Materials

For the assembly of the fiber/LC composites, LC was used as purchased from a local supermarket. CA (Mn = 30000 g mol^−1^) was obtained from Sigma–Aldrich and used without further purification. Acetone (technical grade 99.5% – Panaska Trading CO) was used in the preparation of the CA solutions that were further electrospun.

### 2.2 Fabrication of Electrospun CA Fibers

**Figure 1:**
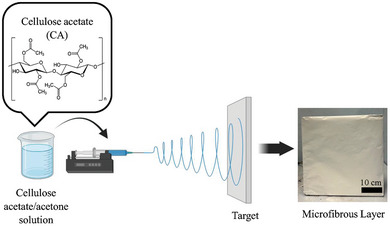
Schematic of the electrospinning process employed in the fabrication of CA electrospun fibrous mats (photograph shown on the right): Step 1: Dissolution of CA in acetone; Step 2: Placement of the polymer solution in the syringe; Step 3: Electrospinning; Step 4: Deposition/collection of the microfibrous layer onto the target.

CA solution was prepared by dissolving CA (1.875 g) in acetone (15 mL). The polymer mixture was left to stir overnight until complete dissolution of the polymer. The resulting colorless transparent homogeneous solution was then loaded in a glass syringe and placed in a custom‐made electrospinning set‐up (**Figure** **1**). All electrospinning experiments were performed at room temperature and at a relative humidity ranging from 40% to 60%. A custom‐made electrospinning set‐up was used, consisting of a controlled‐flow, four‐channel volumetric microdialysis pump (KD Scientific, Model: 789252), a syringe with a connected spinneret (needle electrode), a high‐voltage power source (10–50 kV), and a custom‐designed grounded target collector placed inside an interlocked Faraday enclosure safety cabinet.

For obtaining continuous, bead‐free fibers, the following electrospinning conditions were employed, in line with previous findings^[^ ^32^, ^33^, ^34^ ^]^: Applied voltage: 15 kV; flow rate: 5.9 mL h^−1^; needle diameter: 16 G; needle‐to‐collector distance: 10 cm.

### 2.3 Fabrication of CA fibers/LC Composites

**Figure 2:**
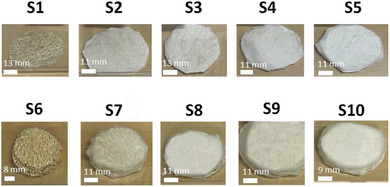
Photographs of the fabricated specimens S1‐S10. Description of their properties and characteristics is provided in Tables 1 and 2.

Two sample series were fabricated by altering the number of the CA microfibrous layers that were wrapped around pristine LC circular specimens. The latter retained a diameter of ≈50 mm, whereas the thickness varied between 3 and 4 mm. In **Tables** **1** and **2**, information on the materials’ composition, thickness, diameter, porosity and density is provided, whereas in **Figure** **2**, the corresponding photographs of all samples are shown. The thickness of all specimens was measured using a digital caliber. Since LC is a natural material, it was not feasible to produce samples having the same thickness. Consequently, the samples were divided into two groups, i.e., the 1st and 2nd sample series. As seen in Tables 1 and 2, within the 1st and 2nd sample series, there is a maximum difference of 5 mm in material thickness, ranging between 3.5 and 9.2 mm in the case of the 1st sample series (Table 1) and between 19.0 and 23.7 mm in the case of the 2nd sample series (Table 2). For this reason, the sound absorption properties were studied separately for each group as described in the following subsections.

**Table 1:** Properties and characteristics of 1st sample series.

Sample code	Structure	Abbreviation	Thickness [mm]	Diameter [mm]	Porosity [%]	Density [Kg m^−3^]
S1	LC	LC	3.5	52	91	48
S2	LC wrapped with a single CA microfibrous layer (**LC/s**)	LC/sCA	4.3	55	94	44
S3	LC wrapped with a double CA layer	LC/dCA	4.8	53	95	32
S4	[LC wrapped with a single CA layer/LC wrapped with a single CA layer] /wrapped with a single CA layer	[LC/sCA]_2_/sCA	8.9	55	94	39
S5	[LC wrapped with a double CA layer/LC wrapped with a double CA layer]/wrapped with a double CA layer	[LC/dCA]_2_/dCA	9.2	55	94	41

**Table 2:** Properties and characteristics of 2nd sample series.

Sample code	Structure	Abbreviation	Thickness [mm]	Diameter [mm]	Porosity [%]	Density [Kg m^−3]^
S6	LC/ LC/LC	[LC/LC/LC]	21.2	41.3	90	44
S7	[LC / LC/ LC]/wrapped with a single CA layer	[LC/LC/LC]/sCA	19.0	42.6	93	42
S8	[LC/LC/ LC]/wrapped with a double CA layer	[LC/LC/LC]/dCA	22.7	44.0	93	43
S9	[LC wrapped with a single CA layer/LC wrapped with a single CA layer/LC wrapped with a single CA layer]/wrapped with a single CA layer	[LC/sCA]_3_/sCA	23.7	44.3	95	40
S10	[LC wrapped with a double CA layer/LC wrapped with a double CA layer/LC wrapped with a double CA layer]/wrapped with a double CA layer	[LC/dCA]_3_/dCA	21.9	46.0	93	46

### 2.4 Morphological Characterization

The morphological characteristics of the as‐received LC, the produced CA fibers and the LC/CA composite structures were determined by Scanning Electron Microscopy (SEM) (Vega TS5136LS‐Tescan, Brno, Czech Republic), with the use of a secondary electron (SE) detector and a voltage of 10 and 20 kV. Prior to their inspection, all samples were gold‐sputtered (sputtering system K575X Turbo Sputter Coater‐Emitech, Quorum Technologies Ltd., West‐Sussex, UK). Cross‐sectional images were obtained by utilizing a cross‐sectional sample holder placed on a tilted stage. The diameters of the LC microchannels and of the CA fibers were measured using an image analysis software (ImageJ).

The surface morphology of the samples was also visualized by confocal microscopy (Olympus LEXT 3100), using a 408 nm laser. The defined displacement of the object in the vertical axis allows a 3D reconstruction of the surface.

### 2.5 Porosity and Density

To gain a more detailed understanding of the structural properties of the samples, measurements were carried out for determining their density and porosity. The density of each sample was determined by calculating its volume and weight after being thoroughly dried. The volume was obtained by multiplying the net cross‐sectional area with the thickness of the sample, ensuring accurate representation of its physical dimensions. Porosity was quantified following the same procedure as described in ref. [35], by assessing the fraction of the sample's total volume that was occupied by pores. Specifically, this was expressed as the ratio of the cumulative pore volume to the overall volume of the sample. The experimental values of porosity and density corresponding to each sample are provided in Tables 1 and 2.

### 2.6 Sound Absorption Coefficient and the Standing Wave Ratio Method

The sound absorption coefficient (*α*) is an important parameter in acoustics, representing the ability of a material to absorb sound energy rather than reflecting it. It measures how much sound energy is dissipated by a material like heat or transformed into other forms of energy. The remaining energy is reflected in the environment. It can be calculated as the ratio between the absorbed energy *E*_a_ and the incident energy *E*_i_ (Equation 1). It is a dimensionless quantity with a value between 0 and 1, where 0 means the material reflects all incident sound energy (no absorption) and 1 means the material absorbs all incident sound energy (total absorption).

(1) $$\alpha =\frac{E_{a}}{E_{i}}\;$$

The sound absorption coefficient plays a critical role in various applications including architectural acoustics,^[^ ^36^ ^]^ noise control in industrial or environmental contexts,^[^ ^37^ ^]^ acoustic panels,^[^ ^38^ ^]^ and device development such as loudspeakers, headphones, etc., where material selection based on absorption properties ensures better sound fidelity or noise isolation.^[^ ^39^ ^]^

The sound absorption coefficient is highly dependent on frequency. A material that absorbs high‐frequency sounds effectively may perform poorly at low frequencies and vice versa. Therefore, absorption coefficients are measured across a range of frequencies, which covers the typical range of human hearing. There are several factors that affect the sound absorption in materials. These include: a) Material thickness: Thicker materials generally absorb better at lower frequencies^[^ ^40^ ^]^; b) Porosity: The more porous a material is, the better its sound absorbing properties will be.^[^ ^41^ ^]^ Porous materials dissipate sound energy as it passes through, converting it into heat.^[^ ^42^ ^]^ c) Surface Texture: The roughness of the surface increases the equivalent acoustic mass, resulting in a larger absorption coefficient at low frequencies.^[^ ^43^ ^]^ d) Mounting: The way a material is mounted or spaced from a hard surface (such as a wall) affects its absorption performance, particularly at low frequencies. For example, an air gap between the material and the wall may significantly improve the low frequency absorption.^[^ ^4^, ^44^ ^]^

The noise reduction effectiveness of multilayer fibrous structures is largely influenced by their configuration. Consequently, various strategies have been developed to enhance their acoustic absorption properties.^[^ ^45^ ^]^ For example, it has been demonstrated that the density of the porous material layers and the perforation ratio of perforated panel layers significantly impact the acoustic absorption of multilayer structures. The outer porous layer should facilitate the easy entry of sound waves, while the inner layers are responsible for effectively dissipating the acoustic energy.^[^ ^46^ ^]^

Currently, both empirical and theoretical models have been developed to describe the acoustic absorption behavior of multilayer materials.^[^ ^47^, ^48^, ^49^ ^]^ However, the accurate prediction of the acoustic absorption coefficient remains challenging, due to the complex mechanisms involved in acoustic energy dissipation. Multilayer fibrous materials, which are composed of different types of fibrous layers, have their noise reduction efficiency affected by both the intrinsic properties of the materials and the sequence in which the layers are arranged.^[^ ^50^ ^]^

The influence of layer arrangement on the acoustic absorption of multilayer nonwoven structures has been examined in polyester nonwoven fabrics. The findings revealed that the noise reduction coefficient increased as the number of layers increased up to three but declined when the number of layers increased to four.^[^ ^12^ ^]^ A combination of polyester/para‐aramid fiber nonwovens and para‐aramid paper achieved higher sound absorption coefficients than glass wool in the mid to high‐frequency ranges. Additionally, fiber assemblies served effectively as an air gap cavity behind the para‐aramid paper.^[^ ^51^ ^]^

Cotton felt underpads significantly enhanced the acoustic absorption of nonwoven fabrics composed of kenaf, jute, and cotton fibers blended with synthetic fibers.^[^ ^52^ ^]^ Sandwich structures, such as those made of PLA/hemp/PLA and PP/glass fiber/PP layers, exhibited improved sound absorption in middle to high frequencies.^[^ ^53^ ^]^

Kucuk and Korkmaz^[^ ^54^ ^]^ developed eight types of bilayered nonwovens using mixed cotton, wool, acrylic, polyester, and PP as base layers, with top layers of polyester or polyester/polyamide nonwovens. They demonstrated that a macrofibrous polyester layer backed with a blend of 70% wool and 30% bi‐component polyester fibers significantly enhanced sound absorption properties.

**Figure 3:**
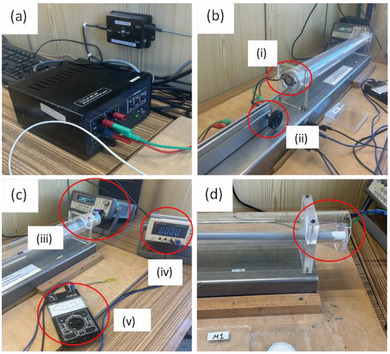
Equipment of the standing wave ratio method. a) Stabilized power source TESLA BK 125. b) Impedance tube mounted at the metal plate with a turning handle system to move the microphone probe within the tube (ii). The speaker is placed on the front surface of the tube (i). c) On the left is the Agilent 33220A arbitrary waveform generator (iii) and on the right and front are the digital (iv) and pointer (v) multimeter accordingly. d) The sample is placed in the tube by removing the lid at the end of the tube.

The sound absorption can be measured either in a reverberation chamber or in an impedance tube. Herein, we applied the method using standing wave ratio in impedance tube that is described in the standard ISO 10534‐1. The experimental set‐up used can be seen in **Figure** **3**. The values are determined by evaluating the standing wave pattern of a plane wave in a tube, which is generated by the superposition of an incident sinusoidal plane wave with the latter reflected from the test object.^[^ ^55^ ^]^ The measurement process requires that each frequency is recorded individually. However, the use of bandpass filtering typically results in a better signal‐to‐noise ratio compared to other measurement methods.^[^ ^56^ ^]^

**Figure 4:**
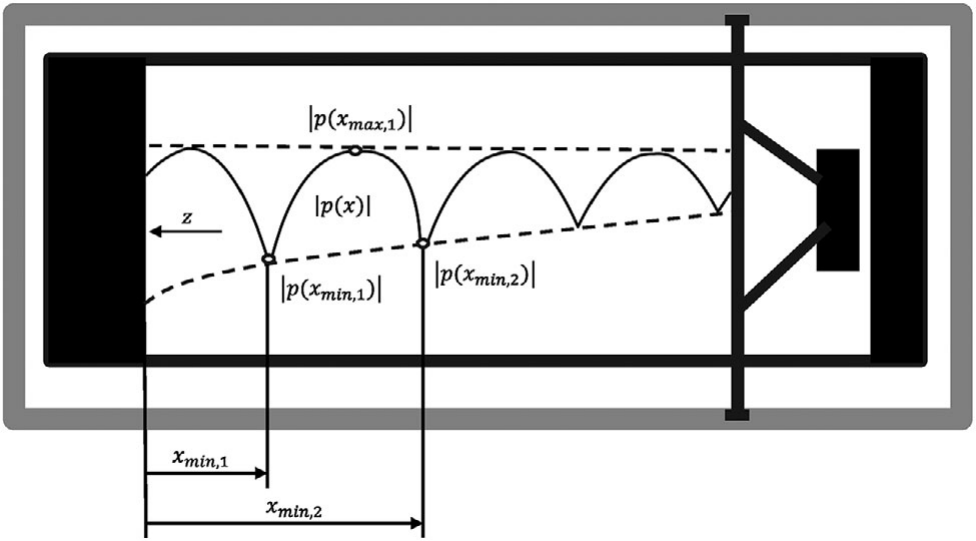
Standing wave pattern in the tube, where *x*_min,1_ is the position of the first minimum pressure *p*_(_x_min,1)_ and *x*_(min,2)_ is the second position that the second minimum pressure *p*_(_x_min,2)_ is measured. In the middle and on the highest point of the sinusoidal plane of these two pressures, the maximum pressure is presented as *p*_(_x_max,1)_. The sample is located on the left side of the tube while the loudspeaker is placed on the right side of the tube.

The Standing Wave Ratio (SWR) method, outlined in ref. [55], is a widely used technique for measuring the sound absorption coefficient and impedance of materials under normal incidence conditions. The method involves the generation of standing waves within an impedance tube, allowing for a detailed analysis of how sound interacts with a material that is placed at the end of the tube. Τhe impedance tube is a rigid cylindrical tube designed to contain sound waves generated by a loudspeaker located at one end. The material under test is positioned at the opposite end of the tube. The tube's dimensions must be carefully selected to ensure that sound waves propagate properly, especially for the desired range of frequencies (**Figure** **4**).

At the beginning of the measurement process, the loudspeaker emits a sound wave that travels through the tube towards the sample. Upon reaching the material, part of the sound energy is absorbed, while the rest is reflected into the tube. The interaction between the incident sound wave (moving toward the material) and the reflected sound wave (bouncing back from the material) creates a standing wave within the tube. Standing waves are characterized by alternating regions of high and low sound pressures, known as pressure maxima and minima, respectively. These patterns are crucial for measuring the reflection properties of the material. The location and intensity of these pressure variations directly relate to how much sound is being absorbed versus reflected by the material. In the SWR method, a movable microphone is employed to determine the ratio between the maximum and minimum sound pressure levels.

Although the SWR method is highly reliable,^[^ ^57^ ^]^ it measures only one frequency at a time, making the process relatively time‐consuming, especially when measuring a large range of frequencies. As previously mentioned, the acoustic performance of the produced materials was investigated by applying the SWR method, following the guidelines of ISO 10534‐1.^[^ ^55^ ^]^ Based on these measurements, the sound absorption coefficient was calculated. The values were obtained by examining the standing wave pattern formed by the superposition of an incident sinusoidal plane wave and its reflection from the test sample inside the tube. Measurements were conducted for the center frequencies of the 1/3 octave bands within the range of 500–4000 Hz (i.e., 500, 630, 800, 1000, 1250, 1600, 2000, 2500, 3150, and 4000 Hz).

The first minimum *p*(*x*_min,1_) and maximum *p*(*x*_max,1_) sound pressures are used to calculate the sound absorption coefficient. According to ref. [55], it is not recommended to measure too close to the sample surface (two times the diameter of the tube). Thus, the other minimum *p(x_min,n_)* and maximum *p(x_max,n_)* of the sound pressures must be used and transformed to the reference plane (the sample surface x = 0). Therefore, Equations (2 and 3) are used to calculate *r* (sound pressure reflection factor at normal incidence):

(2) $$\left|r\right|=\frac{s_{n}e^{k_{o}x_{\min,n}}-e^{-k_{o}x_{\max,n}}}{s_{n}e^{-k_{o}x_{\min,n}}+e^{-k_{o}x_{\max,n}}}$$

where *k_0_”* is the attenuation constant in nepers per meter, *x*_min,n_ is the position of the n^th^ minimum pressure, *x*_max,n_ is the position of the n^th^ maximum pressure and *s*_n_ is the standing wave ratio with attenuation (standing wave ratio of the n^th^ maximum pressure to the n^th^ pressure):

(3) $$\;s_{n}=\frac{\left|p\;\left(x_{max,n}\right)\right|}{\left|p\;\left(x_{min,n}\right)\right|}$$

The sound absorption coefficient (*α*) is then calculated according to Equation (4):

(4) $$\alpha =1-{\left|r\right|}^{2}$$

## 3 Results and Discussion

### 3.1 Morphological Characterization

**Figure 5:**
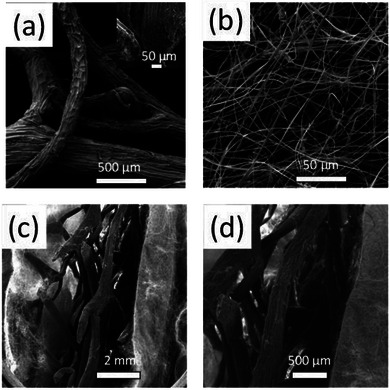
a) SEM images of pure LC used as a sound absorption substrate in both sample series. Magnification image shows its microchannel internal structure. b) SEM image of CA electrospun microfibers. (c‐d) SEM images (cross‐section images) of sample S2 (LC/sCA).

The morphology of LC was initially investigated by SEM. In **Figure** **5**a, characteristic SEM images of LC at various magnifications are exemplarily shown. Cross‐section images reveal a highly porous microchannel morphology with channel diameters ranging between 160 and 400 µm, in line with previously reported findings.^[^ ^58^ ^]^ This 3D highly porous structure of LC renders this material extremely interesting in the development of sound absorbing materials.^[^ ^4^, ^59^ ^]^

The as‐prepared electrospun CA fibers were also visualized by SEM (Figure 5b) that revealed the existence of randomly oriented fibers having a ribbon‐line morphology. This observation agrees with previously reported findings of our group^[^ ^32^, ^33^, ^34^ ^]^ and of other researchers.^[^ ^60^ ^]^ Moreover, the produced fibers are characterized by a relatively broad diameter distribution, ranging between 0.6 and 4.8 µm.

Cross‐sectional SEM images corresponding to sample S2 (LC/sCA) (Figure 5c,d) verified the co‐existence of LC and CA electrospun fibers within a combined LC/CA configuration comprising of LC wrapped with a single CA layer.

**Figure 6:**
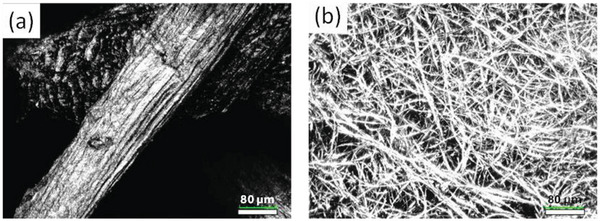
a) Confocal microscopy image recorded for pure LC. b) CA microfibers surface image obtained by confocal microscopy. The scale of each image is indicated at the bottom right. Scale bar: 80 µm.

Confocal microscopy further verified the structure of LC and the CA microfibers (**Figure** **6**). As seen in Figure 6a, the pure LC sample consists of relatively thick LC fibers while large gaps exist between them. The diameter of the luffa fibers was in the order of tens to hundreds of micrometers.

In contrast, the CA microfibers have a diameter in the order of a few µm, in agreement with the SEM findings and the pores existing between them are relatively small. Consequently, the combination of these two materials is considered to be highly advantageous in terms of sound absorption.

### 3.2 Sound Absorption Performance

In terms of sound absorption, from all tested samples, the worst performance was observed in the case of the pure LC (sample S1, LC). Upon increasing the number of LC layers and thus increasing the thickness of the sample (sample S6), the acoustic properties were improved. Further improvement was achieved by incorporating CA electrospun microfibrous layers within the multilayer structures. The obtained sound absorption coefficient values corresponding to each system are summarized in **Table** **3**.

**Table 3:** Sound absorption coefficient values of the sample series.

Sound absorption coefficient [‐]											
Samples		Frequency [Hz]									
		500	630	800	1000	1250	1600	2000	2500	3150	4000
1st sample series	**S1**	0.04	0.05	0.05	0.05	0.05	0.07	0.09	0.10	0.10	0.10
	**S2**	0.05	0.06	0.06	0.08	0.09	0.13	0.17	0.25	0.30	0.35
	**S3**	0.05	0.05	0.06	0.06	0.07	0.09	0.13	0.20	0.24	0.26
	**S4**	0.07	0.08	0.10	0.12	0.15	0.23	0.35	0.55	0.69	0.90
	**S5**	0.06	0.11	0.14	0.20	0.23	0.42	0.58	0.66	0.78	0.81
2nd sample series	**S6**	0.17	0.20	0.17	0.20	0.15	0.19	0.22	0.32	0.43	0.46
	**S7**	0.16	0.22	0.18	0.22	0.21	0.31	0.41	0.59	0.75	0.79
	**S8**	0.19	0.25	0.28	0.34	0.37	0.54	0.71	0.88	0.90	0.82
	**S9**	0.31	0.34	0.37	0.44	0.47	0.63	0.78	0.95	0.97	0.90
	**S10**	0.41	0.45	0.54	0.61	0.71	0.86	0.97	0.97	0.94	0.89

**Figure 7:**
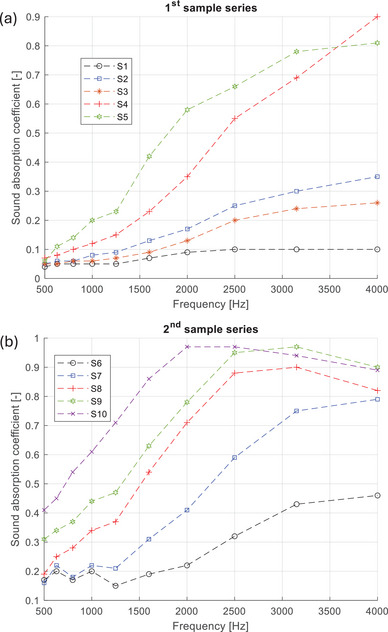
a,b) Sound absorption coefficient (α) versus frequency f (Hz) plots, corresponding to samples belonging to the 1st sample series (a) and the 2nd sample series (b), the description of which is provided in Tables 1 and 2.

Average sound absorption coefficient is the average of the sound absorption coefficients at all ten 1/3 octave frequencies ranging from 500 to 4000 Hz. Average sound absorption coefficients were calculated to easily compare the acoustic performance of all tested samples. In **Table** **4**, the samples are ranked from best to worst in terms of sound absorption for each sample series. The frequency dependence of the sound absorption coefficient is plotted in **Figure** **7**.

**Table 4:** Average sound absorption coefficient for the frequency range 500–4000 Hz.

Average sound absorption coefficient [‐]		
1st sample series	S5	0.40
	S4	0.32
	S2	0.15
	S3	0.12
	S1	0.07
2nd sample series	S10	0.73
	S9	0.62
	S8	0.53
	S7	0.38
	S6	0.25

As seen, all samples exhibit low adsorption at low frequencies, which increases gradually, reaching its maximum between 2000 and 4000 Hz (depending on the sample composition). At lower frequencies, sound waves have longer wavelengths that do not interact as effectively with the closely spaced fibers of the material. Instead, they tend to pass through the material more easily, resulting in lower absorption. Essentially, the fibrous structure is less capable of dissipating the energy of these long waves due to insufficient frictional interaction. In contrast, higher frequency sound waves have shorter wavelengths that are more readily absorbed by the fibrous structure because they interact more frequently with the fibers. The repeated interaction and scattering of these shorter waves within the material cause more significant energy loss through friction, leading to higher sound absorption.

While the sound absorption of the samples consisting of pure and untreated LC was average, the addition of CA layers resulted to its dramatic increase. As seen in the graphs provided in Figure 7a,b, plotting the sound absorption coefficient as a function of frequency for both sample series, sample S10 consisting of three layers of LC each one wrapped with a double CA layer, and all together encapsulated within a double CA layer, (i.e., [LC/dCA]_3_/dCA), demonstrated the best sound absorption performance from all tested samples. By comparing the sound absorption coefficient value recorded for the best‐behaved material (S10) with those reported for a variety of synthetic electrospun micro‐ and nanofibers including PU, nylon and polyacrylonitrile,^[^ ^16^ ^]^ it becomes obvious that the former exhibits comparable and in many cases superior sound absorption performance, while its natural, eco‐friendly character further highlights the added value of naturally‐derived sound absorption materials. As previously stated, the introduction of CA microfibrous membranes characterized by large specific surface area and high porosity^[^ ^26^ ^]^ combined with the increased material's thickness,^[^ ^61^, ^62^ ^]^ resulted in an effective absorption of the sound waves. Concerning the latter, by increasing the thickness of the material, small air gaps are introduced between the layers, which in turn influences the sound absorption coefficient.^[^ ^63^ ^]^ Based on the experimental data, it has been confirmed that thickness is a very important factor in sound absorption performance, particularly in the low‐frequency range.^[^ ^64^ ^]^ The addition of several thin CA microfibrous layers between and on top of the pure LC layers significantly improves the acoustic properties, which might be attributed to the different dimensions and morphology of the CA microfibers and the LC fibers exhibiting a microchannel internal morphology, and the air gabs existing between them.

By comparing the LC/CA composites to synthetic foams such as PU and polystyrene,^[^ ^65^, ^66^ ^]^ distinct advantages of the former can be underlined, including their sustainability, deriving from the fact that they originate from a natural, renewable resource, biodegradability, cost‐effectiveness and comparable or even superior sound absorption performance, deriving from the interconnected pore structure of LC and the high porosity and large surface area derived from the electrospun microfibrous membrane counterpart, promoting enhanced sound dissipation and good performance especially in the high‐frequency region.

Moreover, based on the comparison of the acoustic properties of the produced eco‐friendly LC/CA materials with other natural materials of various thicknesses, including raw natural fibers, fiber assemblies and composites,^[^ ^64^ ^]^ it can be concluded that the former exhibit comparable and, in some cases, superior sound absorption properties. While pure LC mats feature a serrated surface and a microporous structure, they do not achieve high sound absorption coefficients when used alone. By comparing the sound absorption performance of samples S6 and S7, it can be concluded that the inclusion of the CA outer layers already improved the sound absorption parameters. A significantly better improvement in acoustic properties occurred when using double a CA layer (S8) or when using several CA layers (see samples S9 and S10). This was also confirmed for the samples of the first (thin) series.

Audio frequency typically ranges from 20 Hz to 20 kHz. The human auditory system is most sensitive to frequencies between 500 and 4000 Hz.^[^ ^67^ ^]^ Additionally, the key frequencies for hearing and comprehending speech fall between 512 and 2048 Hz.^[^ ^68^ ^]^ As can be seen from Figure 7, sample S10 demonstrated the best performance in that frequency range. From this point of view, it would be desirable to improve the absorption of the samples tested at lower frequencies. One way to achieve this is by including air gaps. Inserting an air gap between the samples and the solid wall could contribute to higher sound absorption coefficient values at lower frequencies and consequently, higher average sound absorption coefficient values. Another possibility is to incorporate graphene into the structure of the CA microfibrous layers, as shown in,^[^ ^28^ ^]^ that could also be the subject of further research.

## 4 Conclusion

This study explored the sound absorption characteristics of CA electrospun fibers/LC composites. The surface of LC fibers is rough and contains numerous porous structures, creating a 3D network that enhances sound energy absorption. The sound absorption properties of the LC fiber composite align with the typical behavior of fibrous sound‐absorbing materials: the sound absorption coefficient is relatively low at low frequencies but gradually increases as the frequency rises (within a specific range). Although pure LC mats have a serrated surface and a microporous structure, they do not exhibit high sound absorption coefficients when used on their own. However, their performance improved significantly when combined with CA electrospun fibers layers due to a reduction in pore size. More precisely, an increase in the average sound absorption coefficient recorded within the frequency range 500–4000 Hz was observed within the 1st sample series, ranging from 0.07 (S1‐pure LC) to 0.4 (S5). Similarly, in the case of the 2nd sample series, the average sound absorption coefficient recorded within the same frequency range varied between 0.25 (S6) and 0.73 (S10). Furthermore, it has been demonstrated that the thickness of the electrospun mats was a significant influencing factor on the sound absorption performance of the produced materials. The combination of LC and double CA layer on top or several CA layers, hold promise for use in architectural settings, where they can help absorb reverberant noise and enhance sound transmission. In summary, the excellent acoustic properties of these materials, combined with their affordability, and environmental friendliness, make them highly appealing for a range of noise and vibration control engineering applications. These mats, being nonhazardous and completely eco‐friendly with adequate sound absorption capabilities, are well‐suited for acoustic applications in buildings and vehicles. Future studies focus on the integration of hollow electrospun fibers within naturally derived composite materials and the incorporation of graphene‐based additives, aiming to improve the sound absorption performance especially at a lower frequency range.

## Conflict of Interest

The authors declare no conflict of interest.

## Author Contributions

T.K.‐C. conceived the idea and designed this project. V.T. performed all experiments and carried out the fabrication of the electrospun fibrous composites. P.P. performed the SEM analysis of the produced samples. M.M., I.K., and I.P. performed all the sound absorption tests and carried out the characterization of the materials by means of confocal microscopy.

## Data Availability

The data that support the findings of this study are available from the corresponding author upon reasonable request.
